# Exploring possibilities for the development of healthy eating habits in preschool settings: parent's and teacher's perspectives

**DOI:** 10.1002/fsn3.3853

**Published:** 2023-12-02

**Authors:** Jelena Niškanović, Dragana Stojisavljević, Stela Stojisavljević, Bosiljka Djikanovic, Dijana Manigoda

**Affiliations:** ^1^ Public Health Institute of the Republic of Srpska Banja Luka Bosnia and Herzegovina; ^2^ Faculty of Medicine University of Banja Luka Banja Luka Bosnia and Herzegovina; ^3^ Institute of Social Medicine, Faculty of Medicine University of Belgrade Belgrade Serbia

**Keywords:** children, healthy eating habits, preschool settings, qualitative study

## Abstract

Adoption of eating habits starts early in life, through interaction with family members and alongside preschool settings that offer context for developing healthy lifestyles among children. The aim of this study was to explore the perceptions and practices of teachers and parents related to the development of healthy eating habits among preschool children. Focus groups were conducted with a total sample of 48 parents and teachers (three focus groups among parents and three among teachers) from 15 kindergartens in the Republic of Srpska, Bosnia, and Herzegovina. All of the mentioned kindergartens are part of the “Nutrition friendly preschool/school initiative”, a program aimed at creating and developing settings that support and improve children's health. Focus groups were audiotaped, transcribed verbatim, and analyzed according to the Framework Method. Teachers emphasized that good communication and support from parents are important factors for the development of healthy eating habits. During COVID‐19, direct communication with parents was restricted, and mutual activities among teachers, children, and parents were reduced. Lack of knowledge, finance, and time are perceived by parents as main obstacles to the improvement of children's healthy eating habits. All participants in the focus groups agreed that more education and informative material are needed so their skills related to good nutrition can be improved and adopted in a culture‐sensitive way. Mutual support, education, and dissemination of informative materials are imposed as particularly important needs by all actors involved in the upbringing of children in order to support the development of children's healthy eating habits.

## Graphical abstract

Adoption of eating habits starts early in life, through interaction with family members and in preschool settings. The aim of this study was to explore the perceptions and practices of teachers and parents related to the development of healthy eating habits among preschool children. Mutual support, education, and dissemination of informative materials are imposed as particularly important needs by all actors involved in the upbringing of children in order to support the development of children's healthy eating habits.

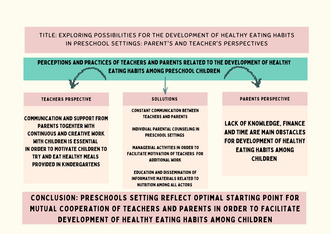

## 1 INTRODUCTION

Consuming healthy food throughout the life span helps to prevent malnutrition as well as a range of non‐communicable diseases (NCDs) and conditions such as diabetes, heart disease, stroke, and cancer (WHO, 2020a). Biological, social, and environmental factors (Ventura & Worobey, 2013) influence the development of food preferences. Early experience serves as the basis for the development of eating habits and their maintenance during later life (Birch & Fisher, 1998; Mahmood et al., 2021), with certain modifications in relation to societal context. At the early stage of life, a child's social context dominantly includes parents and family, which are influenced by community, society, and media (Cuellar et al., 2015). Parents provide culturally sensitive environments and experiences with food and eating for their children, modeling their eating behaviors, lifestyles and eating‐related attitudes (Ogden, 2014; Scagliano et al., 2018; Story et al., 2002). On the one hand, as providers of the food that children eat, family members are also relevant role models and establish rules and norms related to food and eating practices, but as children grow and start preschool and school, teachers, peers, and other people in their social context, together with the media, become more important (Cusatis & Shannon, 1996).

As children grow, their nutrition habits evolve over time, being influenced by many social and economic factors that interact in a complex manner and form individual dietary patterns (WHO, 2020a, 2020b). Therefore, promoting a healthy food environment – including food systems that promote a diversified, balanced, and healthy diet – requires the mutual cooperation of multiple sectors and stakeholders (WHO, 2020a). The most common place for health promotion among children has previously been in the school setting, mostly with children aged 6–12 years‐old (Rito et al., 2020). But there are promising findings in interventions targeting infants and 5‐year‐olds (Brand et al., 2017; Halbeisen & Walther, 2021; Kostecka, 2022; Mikkelsen et al., 2014; Wang et al., 2022). Most of these interventions have been taking place in preschool settings, such as kindergartens, which is very important, especially if we consider that in the mentioned setting, children consume a large number of their meals and may consume up to 70% of their daily nutrient intake (Mikkelsen, 2011; Mikkelsen et al., 2014).

According to the latest studies conducted in the Republic of Srpska, Bosnia and Herzegovina overweight is one of the most dominant issues related to nutritional status of children (MICS, 2013) while, considering the general population, more than half of the inhabitants do not regularly consume tree meals during the day and one in ten add salt to food before tasting it (Matovic Miljanovic et al., 2011). Regular fruit and vegetable consumption on a daily basis is present only among 8.7% (for fruits) and 8.3% (for vegetables) inhabitants (Stojisavljevic et al., 2015). There is a present lack of more recent survey data related to nutrition among the general population, especially among preschool and schoolchildren in the Republic of Srpska, stressing the importance of exploring nutrition and eating habits and their underlying factors among children. Considering the consequences of overweight and obesity on both a personal and societal level and the lack of adequate nutritional habits among the general population, healthier nutrition should be promoted from an early age as one of the actions to prevent overweight and obesity in future generations. The program “Nutrition friendly preschool initiative” (NFPI) has been implemented in the Republic of Srpska (RS), Bosnia and Herzegovina (BiH) since 2014, and it includes 15 public kindergartens from different geographical parts of the RS. The program has been developed and implemented according to the World Health Organization (WHO) “Nutrition‐Friendly School Initiative” (NFSI) (WHO, 2020b), with the aim of developing and facilitating settings that support and improve children's health. NFSI targets schools and preschool setting, and consists of five key components: preschool nutrition policies, awareness and capacity building of the school community, nutrition and health‐promoting curricula, a supportive school environment for good nutrition, and supportive school nutrition and health services (WHO,  2020b). Since its beginning, NFPI in RS has been mainly focused on preschool settings since they provide collective nutrition, care, and prevention in health for children from 6 months to 6 years old, according to the Low on preschool upbringing and education (Official Gazette, 2015, no. 79/15). A broad range of defined criteria within the NFPI's five key components served as a platform to define guidelines and education related to nutritional standards and norms for preschool settings in order to provide balanced and healthy meals for children (Stojisavljević et al., 2016 *)*. Since there is a lack of recent studies related to the nutritional habits of t2020bhe population of RS and considering the importance of family and preschool settings in the development and maintenance of children's eating habits, focus groups were conducted among parents and teachers from kindergartens included in the NFPI in order to in‐depth explore their perceptions and practices related to children's nutrition. The objective of this article is to present the results of a qualitative study and discuss them in order to better understand the interaction and dynamics among factors important for early childhood nutrition practices and to provide recommendations for future interventions in this field.

## 2 KEY MESSAGES

- Preschools setting seems like an optimal starting point for the mutual cooperation of teachers and parents in order to facilitate the development of healthy eating habits among children
- Organization of the working process in preschool settings due to COVID‐19 and lack of motivation for unpaid work among teachers seem to be the main barriers to improving cooperation with parents
- Parental approaches to the development of eating habits among their children are under the influence of practical considerations, such as financial resources, lack of time, and skills related to the preparation of healthy meals
- There is a need to consider activities that will support teachers and parents with additional education and facilitate cooperation in order to improve the capacities of preschool settings for the development of healthy eating habits among children.

## 3 METHODS

### 3.1 Study settings and design

The present study is part of activities conducted within the program “Nutrition friendly preschool initiative” (NFPI) during 2021 and 2022. Focus groups among the 15 kindergartens included in the NFPI were primarily conducted in order to gain insights for improving the ongoing phase of the NFSI, so the presented results are a part of a qualitative study. NFPI has been conducted in RS since 2014 in order to establish and expand a network of kindergartens that promote and improve children's health according to a comprehensive WHO framework (WHO, 2020b). In order to explore parents and teacher perspectives and interactions related to the development of children's nutrition habits in the context of NFPI in the Republic of Srpska, BiH, a qualitative research approach has been used, and data were analyzed according to the Framework Method. Qualitative methods are valuable because the richness of qualitative data permits in‐depth examination of nuances and contradictions, as well as understanding the subjective meanings that people give to their experiences that give rise to certain behaviors (Pistrang & Barker, 2013).

### 3.2 Participants

Participants of the study were recruited from each public preschool (*n* = 15) facility included in the “Nutrition‐friendly preschool initiative” in the Republic of Srpska, BiH. A qualitative study was conducted at the beginning of the 6th phase of NFPI, so 10 preschools were previously involved in NFPI, while 5 preschools were included in the initiative for the first time and did not have any previous experience related to nutrition programs. Participants in focus groups were (1) teachers who work with preschool children in kindergartens and (2) parents of preschool children.

All participants were approached by members of the study team in close cooperation with the management of preschool institutions, who informed target groups face‐to‐face about the study and recruited participants based on the following criteria. The criteria for selecting teachers for participation in the focus group followed the principle of the shortest and longest working experience, thus avoiding potential bias related to professional engagement/experience in preschool settings. Parents were recruited on a voluntary basis and among the participants were predominantly female parents with similar educational status. All participants purposefully participated in the focus groups without incentive.

Separate focus groups among parents and teachers were conducted at each of three geographical locations in the Republic of Srpska, with an expectation of 12 participants per parental focus group and 10 participants per teacher's focus group. The initial number of focus groups and participants who were invited to participate in the focus groups was based on the size of the preschool institutions that were included in the NFPI program (15) and the recommendation from the literature that the optimal number of participants in the focus group is 8–12 (Krueger & Casey, 2000). At the end of the study, six focus groups were conducted (tree among teachers and tree among parents) with a total sample of 48 participants. Response rates for teachers were 96.67% and for parents were 52.78% (Table 1). During the initial contact, all invited parents confirmed their participation in the focus group, while the reason for their non‐appearance was unknown to the researcher. One teacher did not attend the focus group due to health reasons. Saturation in the focus group with teachers was reached during the second focus group, and with parents during the third focus group. The researcher team had the possibility of increasing the number of focus groups in case saturation was not reached, which turned out to be unnecessary.

**TABLE 1:** Number of participants in focus groups by gender, level of education, and location, Republic of Srpska, BiH.

	Teachers	Parents	Total
Sex			
Female	25	13	38
Male	4	6	10
Level of education			
Secondary education	–	9	9
Higher education	29	10	39
Location of focus group			
Banja Luka	10	4	14
Trebinje	8	8	16
Zvornik	11	7	18
Total	29	19	48

### 3.3 Data collection

Before conducting the focus groups, the moderator of the focus group, in cooperation with the coordinator of the “Nutrition‐friendly preschool initiative” (NFPI), prepared a guide with questions for conducting the focus groups. The framework of WHO NFSI has been used for the development of questions according to four broad thematic components: (1) awareness and capacity building of the preschool/ community; (2) nutrition and health promoting curricula; (3) supportive preschool environment for good nutrition; and (4) supportive preschool nutrition and health services. Questions were verified by the program coordinator and adopted for each target group of the study.

Data collection was conducted during May 2021 among participants delegated from 15 public preschool facilities included in the NFPI program, located in cities and municipalities with different levels of development according to the criteria defined by the Government of RS: 9 facilities from developed cities/municipalities, 2 from medium‐developed, 3 from undeveloped, and 1 from extremely underdeveloped municipalities (Gazette, 2019, no. 88/19). Considering that preschool facilities are located in different parts of the Republic of Srpska, focus groups were organized in three cities, so participants were able to come to the closest location.

Before conducting the focus group, the participants were familiarized with the aim and method of conducting the focus group. Participation was voluntary and anonymous. The focus groups were led by an experienced moderator who facilitated the interaction between the participants so all had an equal opportunity to express their views and beliefs. Prior to the study commencement, the moderator was not involved in previous phases of the NFPI program or activities within preschool facilities. During the focus group, the moderator asked participants open questions and encouraged discussion. The shortest focus group lasted 1 h and 11 min, and the longest focus group lasted 1 h and 57 min.

### 3.4 Data analysis

All interviews were recorded, transcribed, and analyzed based on the Framework method (Gale et al., 2013). The main units of research were transcripts, notes, and audio records. Audio records of all focus groups were transcribed verbatim by D.M.

Qualitative data analysis was done by S.S. and D.S. separately in an iterative process that consisted of reading the transcripts and identifying main themes and categories until a stable coding framework was achieved, in the consensus of both researchers. Among the defined main themes, different categories of answers/subthemes were detected (described in Table 2 in detail). In the next phase, transcripts were coded, and researchers compared assigned codes and harmonized them in order to achieve agreement in the definition of categories and further interpretation of the data. Disagreement on one code was discussed with a third researcher (J.N.) to reach consensus. Data interpretation was supported by presenting representative quotes for each finding that were translated into English for the purpose of this paper.

**TABLE 2:** Themes and categories of answers among different groups of participants.

Theme	Categories of answers among different groups of participants
Perception of the role of preschool institutions in the health education of children	Lack of awareness of educational work in preschool institutions (TCH/PAR) Challenges of modern parenthood in relation to preschool settings (TCH/PAR)
The importance of cooperation between the preschool institution and parents	Lack of human resources limits the cooperation between preschool institutions and parents (TCH) Changed cooperation with parents due to the COVID‐19 pandemic (TCH/PAR)
Attitude toward healthy nutrition	Self‐assessment of knowledge about healthy nutrition (TCH/PAR) Critical attitude toward children's nutrition habits (TCH/PAR) The financial costs of healthy nutrition (PAR)
The influence of the modern way of life on family dynamics	Parenting is not easy (TCH/PAR) Parental mistakes based on ignorance or lack of awareness (TCH/PAR)

### 3.5 Ethics

A qualitative study was conducted as one of the activities within the program “Nutrition friendly preschool initiative” in Republic of Srpska, BiH. The initiative was approved by the Ministry of Health and Social Welfare of the Republic of Srpska and the Ministry of Education and Culture of the Republic of Srpska, and supported by UNICEF BiH. Participation in focus groups was voluntary and anonymous. All participants signed a voluntary participation form and gave their consent for recording.

## 4 RESULTS

The analysis of the transcripts of all focus groups revealed four dominant themes: (1) perception of the role of preschool institutions in health education; (2) importance of cooperation between the preschool institution and parents; (3) attitude toward proper nutrition; and (4) influence of the modern way of life.

Each theme has different categories of answers that dominate among study participants. Table 2 provides a list of dominant themes and a summary of each subtheme/category of answers among teachers (TCH) and parents (PAR). In further interpretation, every theme is reported below and elaborated with illustrative quotes for each target group of the study.

### 4.1 Perception of the role of preschool institutions in the health education of children

#### 4.1.1 Perception of teachers

Teachers perceive that preschool institutions have multiple roles; one segment of their work is childcare, but educational work has a greater and more significant role, and it usually takes place through play. They emphasize that they constantly struggle with the opinion of most parents, but also of other actors in the community, that they are “aunts who take care of children” and they have to invest a lot of effort and energy in explaining their role in children's development.

Teachers point out that despite the daily work with parents, organized parental meetings, and consultations, parents still ask more often what the child ate and whether he slept than what he learned in kindergarten. They also state that a small number of parents show no interest at all in what their children do during their stay at the kindergarten and that they often oppose the educators.

When we have meeting with parents, some parents just roll their eyes and can't wait to leave, they weren't interested. (TCH).

It happened that the parents did not know that the child had a meal. I told the mother to wait for the child to eat, and she (the mother) was surprised that the child had to eat in the kindergarten. I was shocked. (TCH).

The teacher still pointed out that the majority of parents are ready to cooperate and are happy to get involved in activities organized by kindergartens. Also, a number of parents, due to working obligations, do not manage to devote enough time to the upbringing and education of their children. These parents have more respect for educator's efforts to develop healthy lifestyle habits among their children. They often say that such support means a lot to them and “that we are both educators and parents”.

#### 4.1.2 Perception of parents

At the beginning, parents perceived kindergartens dominantly as places for childcare. Precisely, they point out that after their child starts to attend kindergarten, they have the opportunity to become familiar with the work of a preschool institution and fully understand the role of teachers in the education of their children.

My children eat too many sweets and in kindergarten they learn that it is not healthy, and now they eat more fruit (PAR).

I am delighted that my child has learned to eat some healthy foods (PAR).

Parents believe that family and parents play a key role in health education and the formation of healthy eating habits. On the other side, some parents believe that educational institutions have an equal role as parents in the health education of their children, especially considering their busy way of life when parents do not have enough time to dedicate to the children.

Some parents also state that they have a hard time dealing with raising their children and that they do not feel sufficiently qualified to cope with everyday parental challenges.

If we want to find time for a child, we will find it, but it is easiest for us to leave the upbringing of the child to someone else, and for us to hide behind the lack of time (PAR).

I don't know what to do with my child, he doesn't know how to play, if he doesn't have a phone he's bored (PAR).

### 4.2 The importance of cooperation between the preschool institution and parents

#### 4.2.1 Teachers

Teachers stressed that achieving a satisfactory level of cooperation with parents demands an investment of a lot of time and effort, and with limited resources, it is quite difficult. As a couple of educators state, preschool institutions must be flexible and appreciate the needs of parents if they want to achieve good cooperation. They pointed out that most parents, due to their regular working activities, are not able to attend parent meetings, workshops, playrooms, and other events that are organized with children in kindergarten during working hours. If activities that involve parents are organized outside of working hours, this implies the additional engagement of teachers on a voluntary basis. In practice, some teachers are more motivated for unpaid overtime work, and some are less, which is reflected in their additional involvement in working with parents.

We have excellent cooperation with parents, we call parents of a certain profession when we organize lectures and workshops, and they help us. We also organize meetings with parents and children, it always gives results (TCH).

It is obvious that we are all different, according to the plan and program we have to hold a meeting with the parents once a year, and whether we will do more, that is now up to us (TCH).

According to educators, COVID‐19 has brought new challenges to maintaining cooperation with parents, and this is especially visible in pre‐school institutions where the number of employees is smaller. In situations where they have to accept or discharge the child from the group, for teachers, it is problematic to leave other children alone in the room, and often they do not have enough time for a quality conversation with the parents.

We feel the difference in work now that there is COVID and before. Before, we had much more communication with parents, we had many workshops, and now we only see parents at the entrance when they hand over their children, it's short, we spend more time walking (TCH).

There aren't many of us in small kindergartens and we have to leave the children alone while we go to receive a child, and then it has to be very fast (TCH).

Educators noted challenges in maintaining cooperation with parents since, due to COVID‐19, they did not have the possibility to organize parent meetings, especially joint workshops with parents and children. They state that they communicated with parents via social networks, by phone, and by placing notices for parents at the entrance to the kindergarten.

#### 4.2.2 Parents

Parents' views are uniform when it comes to cooperation with educators and other staff in preschool institutions. They state that they are satisfied, that they maintain regular contact with the educators, and that they receive enough feedback about what the children are doing in the institution and how their development is progressing. They confirm the educators' statements that direct contacts during the COVID‐19 pandemic were reduced to a minimum, but there is still regular communication with the educators in the institution, especially via social networks such as parental Viber groups.

In this situation, we are more connected than we were before, either through a Facebook group, or by written form, we have letters that child bring home from the kindergarten (PAR).

### 4.3 Attitude toward healthy nutrition

#### 4.3.1 Teachers

Educators with longer work experience state that they are aware that some of the principles of proper nutrition are changing and that they need additional education in order to understand why some foods move from the desirable group to the undesirable group and vice versa (i.e., oil and fat). Younger educators state that during their education they “heard” about proper nutrition and that they have basic theoretical knowledge that needs to be updated and applied in practice.

We are constantly educating ourselves, but we cannot know everything when it comes to nutrition (TCH).

We educators should learn a lot more about healthy eating, we are the ones who teach and take care of children every day (TCH).

Educators state that with the pyramid of healthy food, they easily understood what and in what proportion children, as well as adults, should eat, and that with the help of the pyramid, they easily introduced to children foods that should be consumed more often and those whose intake should be reduced.

With the help of the pyramid, we know how to classify foods, from least to most healthy, and to lay the foundation of nutrition (TCH).

Regardless of the personal self‐assessment of knowledge through discussion, all educators repeatedly mention that the concept of proper nutrition is very broad and includes, among other things, the culture of eating that educators promote. They emphasize that proper nutrition also has to consider rituals related to meals (when a child eats, chews, and sits at the table).

Today, it's important to make children aware of how they eat, so that they don't eat with a mobile phone in their hand, they just yawn and don't look at what they're eating (TCH).

Regardless of the estimated level of knowledge, almost all educators agree that they need additional education, which should be continuous and organized by experts.

Educators and teachers need to be further educated because they can significantly influence children's attitudes (TCH).

#### 4.3.2 Parents

While one group of parents states that they consider their knowledge about healthy eating to be satisfactory, another group of parents observes that they cannot properly assess their knowledge. Despite certain acquired knowledge, parents do not have enough capacity to deal with their children's eating habits.

My kids eat too many candies. (PAR)

Children go to the bakery, take croissants, and sit in front of laptops and cell phones.

Regardless of the estimated level of knowledge about proper nutrition, most parents state financial aspects of healthy nutrition. They claim that foods, which are healthy, are expensive.

We think that healthy eating requires a lot of financial resources (PAR).

However, a few parents disagree with the previous statements that financial reasons dictate the way of eating, arguing that eating habits are determined by everyday lifestyle and insufficient knowledge for applying proper nutrition.

Finances are not an obstacle to proper nutrition, but preparing healthy food requires time, it is easier to buy pâté than to make homemade (PAR).

Today, as parents, we cook less than our mothers did, it's easier to buy “pita” and yogurt and give it to the child for lunch (PAR).

They believe that parents should be taught what proper nutrition is and how they can prepare healthy and affordable meals.

Parents should be taught what proper nutrition is, to what extent children should eat sweets, chips, and not just to forbid everything. (PAR)

### 4.4 The influence of the modern way of life on family dynamics

#### 4.4.1 Teachers

Teachers state that parents are less involved in raising their children, but they find justification for this in the parents' busyness, insufficient knowledge, or lack of parental awareness. They state that parents often make mistakes because they want to please their children and that permissive parenting unconsciously supports undesirable behaviors in children.

Parents give candies to children before they comes to kindergarten, to avoid children's crying, and hungriness, but they have no idea what they did to their child, that they gave him sugar and that the child won't want to have breakfast (TCH).

Educators also notice that the number of children with certain developmental difficulties is constantly rising, and they explain this phenomenon with the excessive use of mobile phones and reduced physical activity:

"There are more and more children between the ages of two and five who arrive to the kindergarten completely non‐verbal, because they spend hours and hours in front of televisions and cell phones." (TCH)

There used to be children who had dysgraphia, but that was rare, and now I notice this problem more and more often, I don't know if it's due to underdeveloped motor skills or some other reason. (TCH)

They state that parents are unknowingly making a mistake by giving their children a phone in order to buy some time for themselves. There is a present need to constantly work with parents on raising awareness of the harmful effects of excessive use of mobile devices.

#### 4.4.2 Parents

According to their statements, half of the parents are very self‐critical, saying that the modern way of life is not to blame for their lack of involvement in raising children. They point out that parents are not able to face existing challenges and claim that the modern way of life has brought modern solutions, but parents, mostly out of ignorance, do not use them or use them in the wrong way. They highlight the example of smart phone technologies that are an irreplaceable source of information and enable parents to quickly gain new knowledge or speed up communication with other parents, educators, and institutions. Less time to spend with their children parents justify with everyday working activates and pressure to provide financial stability for their family, together with too much smart phone usage. Some parents even admitted that everyday smart phone usage steals their time and attention from quality parenting with their children.

We say we don't have time, but in fact we do. We should look at how it was for our mothers who had many more children, they worked much more, and now technology has replaced many things, our mothers didn't have washing machines and dishes, we just run somewhere and hurry, forget the children. (PAR)

If we want to find time, we find it, but it's easiest for us to hide behind the lack of time. (PAR)

Almost all parents point out that today's children start using phones, tablets, and computers too early and that they are aware of their parental responsibility, but that they do not know how to solve the existing challenge.

They don't know how to play, if they don't have a phone they're bored. (PAR)

## 5 DISCUSSION

The findings from this qualitative study identified the perspectives of teachers and parents on different factors that influence the development of children's eating habits. Preschool settings have an important educational function and serve as places that provide quality nutrition and adequate physical activity as essential to growth and development (Harvey‐Berino & Rourke, 2003; McGarvey et al., 2004; Wang et al., 2022), and this aspect seems to be more recognized among participants of pre‐school institutions that have a proactive approach, such as promotion of their programs and activities in the local community (Chaudhary et al., 2020; Nyberg et al., 2011; Wang et al., 2022). Parents who stated they do not have enough time to devote it to the upbringing and education of children more appreciate educator's efforts to develop healthy lifestyle habits among their children. Studies of different interventions in preschool settings revealed that the educational component is important for facilitating vegetable consumption (Mikkelsen et al., 2014), so for the development of proper nutrition, educational work in preschool settings can be beneficial, not just for children but also for parents (Rubu et al., 2014).

A lot of activities related to proper nutrition in preschool settings were implemented from 2014 through the NFPI in the Republic of Srpska, such as the development and adoption of nutrition standards and norms with a cookbook for preschool institutions (Stojisavljević et al., 2016) and a rulebook that defines the area of nutrition, care, health, and social protection of preschool‐age children (Official Gazette, 2016, no. 88/16), so educators and parents became more aware of the importance of healthy meals that children had the opportunity to consume in kindergartens. Since preschool facilities started to adopt meals according to nutrition standards and norms, children have become more familiar with the healthy food items to which they are exposed (Hill, 2002; Ventura & Birch, 2008). Other important aspects of kindergartens are educational components that provide opportunities for the development of healthy eating habits (Mikkelsen et al., 2014) through mutual cooperation among teachers and parents. Cooperation with parents is one of the most important aspects of kindergartens everyday functioning, but it is determined by the motivation of teachers and the COVID‐19 situation. During COVID‐19, direct communication with parents was restricted, and mutual activities among teachers, children, and parents were reduced. So, the educational potential of kindergartens was diminished and it seems more important if we consider that COVID‐19 changed everyday feeding routines by increasing the amount of unhealthy food in homes (Adams et al., 2020; Pujia et al., 2021). Resolving the mentioned obstacles, through transparent online communication, individual parental counseling in preschool settings, and managerial activities in order to facilitate the motivation of teachers (Ekwaru et al., 2017; Kesztyus et al., 2017) seems to be important for providing context for better mutual interaction between teachers and parents that can facilitate the development of healthy eating habits among children.

Multiple factors influence dietary habits and are reciprocally interacting, so they cannot be viewed separately. The family system that surrounds a child's domestic life will have an active role in establishing and promoting behaviors that will persist throughout his or her life (Scagliano et al., 2018). Most of the parents stressed that the everyday way of life with a lot of working activities and constant mobile usage reduces their time with children, which can be managed in a quality way. Lack of time, financial resources, and knowledge needed for the preparation of affordable and healthy food are the main reasons for the lack of an everyday healthy nutrition routine, stressing the importance of socio‐economic factors for the development of dietary patterns (Gutierrez‐Camacho et al., 2019). Similar results of studies conducted among low‐income families have highlighted the relationship between restricted budgets and food choices, resulting in the lower possibility of consuming fruit and vegetables (Hayrel et al., 2015) and Ziauddeen et al. (2018) stressed that nowadays families prepare less food at home and spend more money on food prepared away from home (Ziauddeen et al., 2018). Also, parents are aware that they do not have enough skills to deal with every parental challenge and to motivate their children to eat healthier and be physically active. A lot of studies claim that family, particularly the parents or other primary caregivers, may influence diet and food preference by modeling and controlling the portion sizes and dietary quality of the food they make available to their children (Davison & Birch, 2001; Mahmood et al., 2021), so parents need education and support in order to develop their parental skills. According to teachers, permissive parenting has been seen as the main reason for unconscious facilitation of unhealthy habits and candy consumption, which was also confirmed among some studies that revealed associations among parenting styles and children's dietary behaviors (Kremers et al., 2003; Lindsay et al., 2018; Pearson et al., 2010). Parents and educators shared concerns related to extensive mobile usage among children, especially educators who associate it with developmental delays and children's eating rituals. Exposure to digital screens may be detrimental for children's physical health, cognitive skills, and psychosocial development (Canadian Pediatric Society, 2017), revealing the importance of a deeper understanding of how modern technology shapes children's everyday life, social interactions, and attitudes toward food consumption.

For a lot of teachers, knowledge about proper nutrition is something that needs to be updated by regular professional education with experts, so their skills can be improved in order to organize quality educational activities with children. Raising parental skills, knowledge, and confidence seems to be very important for parents in order to improve healthier habits among their children (Lindsay et al., 2018). Preschool settings seem to be a valuable starting point for mutual cooperation among teachers and parents in order to create a stimulating context for the development of healthy eating habits among children while they are young (Campbell & Crawford, 2001). Successful cooperation and interventions can be achieved by taking into account parents knowledge and intentions and by working with parents in order to reconcile the gap between knowledge and intentions with their daily routines and difficulties in parenting and working activities. In line with key NFPI components, there is space to improve and conduct activities that target as many levels as possible, including interpersonal, social and cultural, environmental, and policy levels.

The findings of the study should be considered bearing in mind some limitations, such as the small sample size and purposive sample of teachers and parents from preschool facilities included in the NFPI program, which limit the generalizability of the results. Parents may have decided to participate in the study due to their interest in NFPI and/or healthy eating, which may further limit generalizability. Nevertheless, the study provides insights into personal perceptions and practices related to the development of children's eating habits among educators and parents in preschool settings located in different geographical parts of the Republic of Srpska, providing a baseline for deeper exploration of the relationship between socio‐economic status and cultural context as factors that shape eating attitudes and habits.

## 6 CONCLUSION

Raising awareness about educational aspect of kindergartens and their role in the development of healthy eating habits among children is one of the most important aspects of the NFPI program. Among proactive kindergartens whose activities become visible in local communities, there is good cooperation with parents and a better understanding of their importance and role in children's development, not only among parents, but also among stakeholders in local community. Better connection, cooperation, and understanding among all key actors are one of the main steps in the development of a context that can facilitate healthy eating habits among children.

## AUTHOR CONTRIBUTIONS

**Jelena Niskanovic:** Data curation (equal); formal analysis (equal); visualization (lead); writing – original draft (lead); writing – review and editing (lead). **Dragana Stojisavljević:** Conceptualization (equal); formal analysis (equal); methodology (equal); writing – original draft (equal); writing – review and editing (equal). **Stela Stojisavljevic:** Data curation (equal); formal analysis (equal); investigation (equal); methodology (equal); writing – original draft (equal); writing – review and editing (equal). **Bosiljka Djikanović:** Supervision (supporting); validation (supporting); writing – review and editing (supporting). **Dijana Manigoda:** Data curation (equal); investigation (equal); project administration (equal).

## CONFLICT OF INTEREST STATEMENT

D.S. is the coordinator of the “Nutrition friendly preschool initiative” in the Republic of Srpska. S.S., J.N., and D.M. are members of the team for the implementation of the “Nutrition friendly preschool/school initiative” in the Republic of Srpska. B.DJ. is an external consultant for qualitative research methodology.

## Data Availability

The data that support the findings of this study are available on request from the corresponding author. The data are not publicly available due to privacy or ethical restrictions.

## References

[fsn33853-bib-0001] Adams, E. L. , Caccavale, L. J. , Smith, D. , & Bean, M. K. (2020). Food insecurity, the home food environment, and parent feeding practices in the era of COVID‐19. Obesity, 28, 2058–2063. 10.1002/oby.22996 PMC7436743 32762129

[fsn33853-bib-0002] Agency for Statistics of Bosnia and Herzegovina, Federal Ministry of Health, Ministry of Health and Social Welfare Republik of Srpska and the Institute of Public Health of the Federation of Bosnia and Herzegovina . (2013). Research on multiple indicators (MICS) for Bosnia and Herzegovina 2011–2012: Final report Sarajevo. UNICEF. https://mics‐surveys‐prod.s3.amazonaws.com/MICS4/Europe%20and%20Central%20Asia/Bosnia%20and%20Herzegovina/2011‐2012/Final/Bosnia%20and%20Herzegovina%202011‐12%20MICS_Bosnian.pdf

[fsn33853-bib-0003] Birch, L. L. , & Fisher, J. O. (1998). Development of eating behavior among children and adolescents. Pediatrics, 101, 539–549. 12224660

[fsn33853-bib-0004] Brand, T. , Jahn, I. , Pohlabeln, H. , Böttcher, S. , Hense, S. , Hebestreit, A. , & Ahrens, W. (2017). Comparing strategies to improve the implementation of healthy nutrition in kindergartens: A prospective observational study. Journal of Public Health, 25(3), 299–310. 10.1007/s10389-016-0779-7

[fsn33853-bib-0005] Campbell, K. , & Crawford, D. (2001). Family food environments as determinants of preschool aged children's eating behaviours: Implication for obesity prevention policy. Australian Journal on Nutrition and Dietetics, 58, 19–25.

[fsn33853-bib-0006] Canadian Pediatric Society . (2017). Screen time and young children: Promoting health and development in a digital world. Pediatrics & Child Health, 22, 461–477. 10.1093/pch/pxx123 29601064 PMC5823000

[fsn33853-bib-0007] Chaudhary, A. , Sudzina, F. , & Mikkelsen, B. E. (2020). Promoting healthy eating amon young people‐ a review of the evidence of the impact of school‐ based interventions. Nutrients, 12, 2894. 10.3390/nu12092894 32971883 PMC7551272

[fsn33853-bib-0008] Cuellar, J. , Jones, D. J. , & Sterrett, E. (2015). Examining parenting in the neighborhood context: A review. Journal of Child and Family Studies, 24, 195–219. 26392738 10.1007/s10826-013-9826-y PMC4573634

[fsn33853-bib-0009] Cusatis, D. C. , & Shannon, B. M. (1996). Influences on adolescent eating behavior. The Journal of Adolescent Health, 18, 27–34. 8750425 10.1016/1054-139X(95)00125-C

[fsn33853-bib-0010] Davison, K. K. , & Birch, L. L. (2001). Childhood overweight: A contextual model and recommendations for future research. Obesity Reviews, 2, 159–171. 12120101 10.1046/j.1467-789x.2001.00036.x PMC2530932

[fsn33853-bib-0011] Ekwaru, J. P. , Ohinmaa, A. , Tran, B. X. , Setayeshgar, S. , Johnson, J. A. , & Veugelers, P. J. (2017). Cost‐effectiveness of a school‐based health promotion program in Canada: A life‐course modeling approach. PLoS ONE, 12(5), e0177848. 10.1371/journal.pone.0177848 28542399 PMC5436822

[fsn33853-bib-0012] Gale, N. K. , Heath, G. , Cameron, E. , Rashid, S. , & Redwood, S. (2013). Using the framework method for the analysis of qualitative data in multi‐disciplinary health research. BMC Medical Research Methodology, 13(1), 117. 10.1186/1471-2288-13-117 24047204 PMC3848812

[fsn33853-bib-0013] Gazette Republic of Srpska . (2019). The decision on the criteria for assessing the level of development of units of local self‐government in the Republic of Srpska. https://www.narodnaskupstinars.net/?q=la/printpdf/19147

[fsn33853-bib-0014] Gutierrez‐Camacho, C. , Mandez‐Sanchez, L. , Klunder‐Klunder, M. , Clark, P. , & Denova‐Gutierrez, E. (2019). Association between sociodemographic factors and dietary patterns in children under 24 months of age. Nutrients, 11, 2006. 10.3390/nu11092006 31454895 PMC6770717

[fsn33853-bib-0015] Halbeisen, G. , & Walther, E. (2021). How to promote healthy eating in preschool children: Evidence from an associative conditioning procedure with non‐food stimuli. Appetite, 166, 105472. 10.1016/j.appet.2021.105472 34153424

[fsn33853-bib-0016] Harvey‐Berino, J. , & Rourke, J. (2003). Obesity prevention in preschool native‐ American children: A pilot study using home visiting. Obesity Research, 11, 606–611. 12740449 10.1038/oby.2003.87

[fsn33853-bib-0017] Hayrel, A. K. M. , Draper, A. K. , Ohly, H. R. , Rees, G. A. , Pettinger, C. , McGlone, P. , & Watt, R. G. (2015). A qualitative study exploring parental accounts of feeding pre‐school children in two low‐income populations in the UK. Maternal & Child Nutrition, 11, 371–384. 23316717 10.1111/mcn.12017 PMC6860273

[fsn33853-bib-0018] Hill, A. J. (2002). Developmental issues in attitudes to food and diet. The Proceedings of Nutrition Society, 61, 259–266. 10.1079/PNS2002152 12133208

[fsn33853-bib-0019] Kesztyüs, D. , Lauer, R. , Kesztyüs, T. , Kilian, R. , Steinacker, J. M. , & on behalf of the “Join the Healthy Boat” Study, G . (2017). Costs and effects of a state‐wide health promotion program in primary schools in Germany – The Baden‐Württemberg study: A cluster‐randomized, controlled trial. PLoS ONE, 12(2), e0172332. 10.1371/journal.pone.0172332 28222101 PMC5319648

[fsn33853-bib-0020] Kostecka, M. (2022). The effect of the “colorful eating is healthy eating” long‐term nutrition education program for 3‐ to 6‐year‐olds on eating habits in the family and parental nutrition knowledge. International Journal of Environmental Research and Public Health, 19, 1981. 35206167 10.3390/ijerph19041981 PMC8872545

[fsn33853-bib-0021] Kremers, S. P. , Brug, J. , de Vries, H. , & Engels, R. C. (2003). Parenting style and adolescent fruit consumption. Appetite, 41, 43–50. 12880620 10.1016/s0195-6663(03)00038-2

[fsn33853-bib-0022] Krueger, R. A. , & Casey, M. A. (2000). Focus groups: A practical guide for applied research. Sage Publications.

[fsn33853-bib-0023] Lindsay, A. M. , Wallington, S. F. , Lees, F. D. , & Greaney, M. L. (2018). Exploring how the home environment influences eating and physical activity habits of low‐income, Latino children of predominantly immigrant families: A qualitative study. Environmental Research and Public Health, 15, 978. 10.3390/ijerph15050978 29757941 PMC5982017

[fsn33853-bib-0024] Mahmood, L. , Flores‐Barrantes, P. , Moreno, L. A. , Manios, Y. , & Gonzales‐Gil, E. M. (2021). The influence of parental dietary behaviors and practice on Children's eating habits. Nutrients, 13, 1138. 10.3390/nu13041138 33808337 PMC8067332

[fsn33853-bib-0025] Matovic Miljanovic, S. , Grozdanov, J. , Bozanic, V. , Bojanic, J. , Stojisavljević, D. , & Šiljak, S. (2011). Health survey among general population of the republic of Srpska. Public Health Institute of the Republic of Srpska.

[fsn33853-bib-0026] McGarvey, E. , Keller, A. , Forrester, M. , Williams, E. , Seward, D. , & Suttle, D. E. (2004). Feasibility and benefits of a parent‐focused preschool child obesity intervention. American Journal of Public Health, 94, 1490–1495. 15333300 10.2105/ajph.94.9.1490 PMC1448479

[fsn33853-bib-0027] Mikkelsen, B. E. (2011). Images of foodscapes: Introduction to foodscape studies and their application in the study of healthy eating out‐of‐home environments. Perspectives in Public Health, 131, 209–216. 21999025 10.1177/1757913911415150

[fsn33853-bib-0028] Mikkelsen, M. V. , Husby, S. , Skov, L. R. , & Perez‐Cueto, F. J. A. (2014). A systematic review of types og healthy eating interventions in preschools. Nutrition Journal, 13, 56. 24906305 10.1186/1475-2891-13-56 PMC4074866

[fsn33853-bib-0029] Nyberg, G. , Sundblom, E. , Norman, A. , & Elinder, A. S. (2011). A healthy school start – parental support to promote healthy dietary habits and physical activity in children: Design and evaluation of a cluster‐randomized intervention. BMC Public Health, 11, 185. 10.1186/1471-2458-11-185 21439049 PMC3071321

[fsn33853-bib-0030] Official Gazette Republic of Srpska . (2015). Low on preschool upbringing and education. https://www.vladars.net/sr‐SP‐Cyrl/Vlada/Ministarstva/mpk/predskolsko/Pages/splash.aspx

[fsn33853-bib-0031] Official Gazette Republic of Srpska . (2016). Rulebook that defines the area of nutrition, care, health and social protection of preschool age children. https://www.vladars.net/sr‐SP‐Cyrl/Vlada/Ministarstva/mpk/predskolsko/Pages/splash.aspxOfficial

[fsn33853-bib-0033] Ogden, J. (2014). The good parenting food guide: Managing what children eat without making food a problem. John Wiley&Sons.

[fsn33853-bib-0034] Pearson, N. , Atkin, A. J. , Biddle, S. J. , Gorely, T. , & Edwardson, C. (2010). Parenting styles, family structure and adolescent dietary behaviour. Public Health Nutrition, 13, 1245–1253. 19954574 10.1017/S1368980009992217

[fsn33853-bib-0035] Pistrang, N. , & Barker, C. (2013). Varieties of qualitative research: A pragmatic approach to selecting methods. In H. Cooper (Ed.), APA handbook of research methods in psychology (Vol. 2, pp. 5–18). American Psychological Association.

[fsn33853-bib-0036] Pujia, R. , Ferro, Y. , Maurotti, S. , Khoory, J. , Gazzaruso, C. , Pujia, A. , Mantolcini, T. , & Mazze, E. (2021). The effects of COVID‐19 on the eating habits of children and adolescents in Italy: A pilot survey study. Nutrients, 13, 2641. 10.3390/nu13082641 34444801 PMC8400531

[fsn33853-bib-0037] Rito, A. I. , Mandes, S. , Santos, M. , Goiana‐da‐Silva, F. , Cappuccio, F. P. , Whiting, S. , Dinis, A. , Rascoa, C. , Castanheira, I. , Darzi, A. , & Bread, J. (2020). Salt reduction strategy in Portuguese school meals, from pre‐school to secondary education‐ the eat Mediterranean program. Nutrients, 12, 2213. 10.3390/nu12082213 32722323 PMC7469016

[fsn33853-bib-0038] Rubu, A. , Messiah, S. E. , Asfour, L. , Uhlhorn, S. , Delamater, A. , & Arheart, K. L. (2014). Role modeling as an early childhood obesity prevention strategy: Effect of parents and teachers on preschool Children's healthy lifestyle habits. Journal of Developmental and Behavioral Pediatrics, 35, 378–387. 25007060 10.1097/DBP.0000000000000074

[fsn33853-bib-0039] Scagliano, S. , de Cosmi, V. , Ciappolino, V. , Parazzini, F. , Brambilla, P. , & Agostini, C. (2018). Factors influencing Children's eating Behaviours. Nutrients, 10(6), 709. 10.3390/nu10060706 29857549 PMC6024598

[fsn33853-bib-0040] Stojisavljevic, D. , Eric Marinkovic, J. , Siljak, S. , Kvaternik, M. , Niskanovic, J. , & Marinkovic, D. (2015). Health risk factors for non‐communicable diseases (NDC) – implications for policy and practice: Survey results for republic of Srpska. Banja Luka: The Public Health Institute of Republic of Srpska [unpublished report].

[fsn33853-bib-0041] Stojisavljević, D. , Stanivuk, L. J. , Todorović, M. , & Petković, V. (2016). Nutrition standards and norms for planning menus in preschool facilities, for children 1 to 6 years old. Public Health Institute Republic of Srpska. https://www.phi.rs.ba/pdf/brosure/standardi%20ishrane%20u%20predskolskim%20ustanovama‐za%20web.pdf

[fsn33853-bib-0042] Story, M. , Neumark‐Sztainer, D. , & French, S. (2002). Individual and environmental influences on adolescent eating behaviors. Journal of the American Dietetic Association, 102, 40–51. 10.1016/s0002-8223(02)90421-9 11902388

[fsn33853-bib-0043] Ventura, A. , & Birch, L. (2008). Does parenting affect children's eating and weight status? International Journal of Behavioral Nutrition and Physical Activity, 5, 15. 10.1186/1479-5868-5-15 18346282 PMC2276506

[fsn33853-bib-0044] Ventura, A. K. , & Worobey, J. (2013). Early influences on the development of food preferences. Current Biology, 23, 401–408. 10.1016/j.cub.2013.02.037 23660363

[fsn33853-bib-0045] Wang, X. , Wu, L. , Liu, Q. , & Wu, Y. (2022). Dietary environment in early care and education settings and young Children's eating behavior: A systematic review of literature. American Journal of Health Behavior, 46, 541–557. 36333831 10.5993/AJHB.46.5.5

[fsn33853-bib-0047] WHO . (2020a). Healthy diet. https://www.who.int/news‐room/fact‐sheets/detail/healthy‐diet

[fsn33853-bib-0048] WHO . (2020b). Nutrition action in schools: A review of evidence related to the nutrition‐friendly schools initiative. World Health Organization.

[fsn33853-bib-0050] Ziauddeen, N. , Page, P. , Penney, T. L. , Nicholson, S. , Kirk, S. F. , & Almiron‐Roig, E. (2018). Eating at food outlets and leisure places and “on the go” is associated with less‐healthy food choices than eating at home and in school in children: Cross‐sectional data from the UK national diet and nutrition survey rolling program (2008–2014). American Journal of Chlinical Nutrition, 107, 992–1003. 10.1093/ajcn/nqy057 PMC5985724 29741556

